# Biogeochemical Mechanisms of HCO_3_–Ca Water and NO_3_^−^ Pollution in a Typical Piedmont Agricultural Area: Insights from Nitrification and Carbonate Weathering

**DOI:** 10.3390/toxics13050394

**Published:** 2025-05-15

**Authors:** Li Xu, Bo Xin, Wei Liu, Haoyang Liu, Guoli Yang, Guizhen Hao

**Affiliations:** 1 School of Energy and Environmental Engineering, Hebei University of Engineering, Handan 056038, China; xuli0031@163.com; 2 Hebei Key Laboratory of Water Quality Engineering and Comprehensive Utilization of Water Resources, Hebei University of Architecture, Zhangjiakou 075000, China; lw_hue@126.com (W.L.); liuhaoyangsz@163.com (H.L.); hgz1193@hebiace.edu.cn (G.H.); 3 Zhangcheng Ecological Environmental Protection and Restoration Technology Innovation Center, No. 3 Geological Brigade of Hebei Geology and Mineral Exploration Bureau, Zhangjiakou 075000, China; xb9968@126.com

**Keywords:** HCO_3_–Ca water, NO_3_^−^ pollution, agricultural ammonium, nitrification, carbonate dissolution, piedmont area

## Abstract

Water hardening and NO_3_^−^ pollution have affected water quality globally. These environmental problems threaten social sustainability and human health, especially in piedmont agricultural areas. The aim of this study is to determine the biogeochemical mechanisms of HCO^3^–Ca water and NO_3_^−^ pollution in a typical piedmont agricultural area (Qingshui River, Zhangjiakou, China). Here, an extensive biogeochemical investigation was conducted in a typical piedmont agricultural area (Qingshui River, China) using multiple hydrochemical, isotopic (δ^2^H-H_2_O, δ^18^O-H_2_O and δ^13^C-DIC) and molecular-biological proxies in combination with a forward model. In the region upstream of the Qingshui River, riverine hydrochemistry was dominated by HCO_3_–Ca water, with only NO_3_^−^ concentrations (3.08–52.8 mg/L) exceeding the acceptable limit (10 mg/L as N) for drinking water quality. The riverine hydrochemistry responsible for the formation of HCO_3_–Ca water was mainly driven by carbonate dissolution, with a contribution rate of 49.8 ± 3.96%. Riverine NO_3_^−^ was mainly derived from agricultural NH_4_^+^ emissions rather than NO_3_^−^ emissions, originating from sources such as manure, domestic sewage, soil nitrogen and NH_4_^+^-synthetic fertilizer. Under the rapid hydrodynamic conditions and aerobic water environment of the piedmont area, NH_4_^+^-containing pollutants were converted to HNO_3_ by nitrifying bacteria (e.g., *Flavobacterium* and *Fluviimonas*). Carbonate (especially calcite) was preferentially and rapidly dissolved by the produced HNO_3_, which was attributed to the strong acidity of HNO_3_. Therefore, higher levels of Ca^2+^, Mg^2+^, HCO_3_^−^ and NO_3_^−^ were simultaneously released into river water, causing riverine HCO_3_–Ca water and NO_3_^−^ pollution in the A-RW. In contrast, these biogeochemical mechanisms did not occur significantly in the downstream region of the river due to the cement-hardened river channels and strict discharge management. These findings highlight the influence of agricultural HNO_3_ on HCO_3_–Ca water and NO_3_^−^ pollution in the Qingshui River and further improve the understanding of riverine hydrochemical evolution and water pollution in piedmont agricultural areas.

## 1. Introduction

River water is an essential fresh-water resource for agricultural irrigation, industrial production, potable water and social development, particularly in piedmont areas, where groundwater resources cannot easily be exploited due to the depth of the groundwater table. Water resource shortages and water pollution are increasingly affecting both urban and rural areas worldwide due to climate stress [1,2], population growth [3], pollution risks [4,5,6] and water conflicts [7,8]. Therefore, water quality problems, such as water hardening and NO_3_^−^ pollution, continue to be a major focus in environmental toxicology research [9,10,11].

A high concentration of NO_3_^−^ in water can result in eutrophication and toxic algal blooms that are harmful to aquatic organisms [12,13], and toxic hazards that are associated with birth defects, cancer and methemoglobinemia via drinking water with high NO_3_^−^ levels have been documented [14,15,16]. Although excessively high concentrations of HCO_3_^−^, Ca^2+^ and Mg^2+^ do not pose a direct toxic effect on humans, lithiasis induced by the crystallization of these ions not only causes great harm to the human body but may even lead to related tissue lesions.

High concentrations of HCO_3_^−^, Ca^2+^ and Mg^2+^ contribute to water hardening, with these ions generally originating from carbonate weathering [17,18]. Mineral weathering involves a series of important and complex hydrogeochemical evolution processes due to the diversity of natural minerals and the mixed states of minerals weathering in natural aquatic systems [19,20]. In theory, carbonate dissolution releases HCO_3_^−^, Ca^2+^ and Mg^2+^ into river water under unsaturated conditions [11,21]. Furthermore, carbon isotope (δ^13^C) of dissolved inorganic carbon (DIC) is a useful tool to carbonate weathering processes. More DIC from carbonate weathering (old carbon) induced by strong acid (e.g., HNO_3_) leads to a higher δ^13^C-DIC level, and more DIC from atmosphere/soil CO_2_ (new carbon) in natural carbonate weathering processes causes a lower δ^13^C-DIC level [22]. Therefore, the involvement of different acids (e.g., H_2_CO_3_ and HNO_3_) in carbonate dissolution can release specific δ^13^C-DIC signals into the water environment [19,23], providing a comprehensive understanding of carbonate weathering. However, previous studies on the formation mechanisms of HCO_3_–Ca water have mainly focused on karst areas, with few investigations conducted in piedmont agricultural areas.

River water suffers from NO_3_^−^ pollution in intensive agriculture regions, and piedmont agricultural areas are no exception. Given the large amount of NH_4_^+^ contained in agricultural emissions [24,25], agricultural NH_4_^+^ generally undergoes complex biogeochemical processes before being converted to riverine NO_3_^−^. Nitrification induced by nitrifying bacteria is an important process driving the nitrogen biogeochemical cycle, which can convert NH_4_^+^ in nature into NO_3_^−^, especially in oxygen-rich water environments with better hydrodynamic conditions. Riverine NO_3_^−^ produced from NH_4_^+^ requires 2/3O-H_2_O and 1/3O-O_2_ via microbial nitrification processes [26,27], which can result in a high level of δ^18^O-H_2_O enrichment in river water due to the isotopic kinetic fractionation effect. Furthermore, high NH_4_^+^ contents contribute to the metabolism of nitrifying bacteria, resulting in a negative correlation with NH_4_^+^ and a positive correlation with NO_3_^−^ [27,28]. Hence, the combined use of hydrochemical, isotopic and molecular biological indicators can provide a reliable and comprehensive understanding of microbial nitrification processes. However, previous reports have focused mainly on assessing the fates and sources of NO_3_^−^ [27,29,30]. It is of note that HNO_3_ can be rapidly produced from agricultural NH_4_^+^ by nitrifying bacteria in aerobic water environments [13], especially in piedmont agricultural areas [28]. The involvement of agricultural HNO_3_ in carbonate dissolution has been verified in karst areas in recent years [13,25,31,32], which has overturned the traditional understanding of karst hydrochemical evolution and provided novel insights into the characteristics of HCO_3_–Ca water and NO_3_^−^ pollution in karst agricultural regions. However, further studies are required to comprehensively understand the effect of anthropogenic acids on mineral dissolution and riverine pollution in piedmont agricultural areas.

The Qingshui River is located in the transitional zone between the Inner Mongolia Plateau and the North China Plain. The river water composition is dominated by HCO_3_–Ca type controlled by the hydrogeochemical evolution in the piedmont area [24,33]. Due to long-term agricultural emissions, widespread diffuse NO_3_^−^ pollution has been found in both the river water and groundwater of the Qingshui River basin [28,34]. Hence, the Qingshui River is a suitable field for determining the biogeochemical mechanisms of HCO_3_–Ca water and NO_3_^−^ pollution. Previous reports have considered carbonate dissolution and agricultural nitrogen emissions as separate hydrochemical processes, which may reduce the accuracy of conclusions on the co-enrichment processes of riverine Ca^2+^, Mg^2+^, HCO_3_^−^ and NO_3_^−^. Furthermore, the influence of agricultural HNO_3_ on the co-formation mechanisms of riverine HCO_3_–Ca water and NO_3_^−^ pollution remains largely unknown in piedmont agricultural areas. To better understand the biogeochemical mechanisms of riverine HCO_3_–Ca water and NO_3_^−^ pollution, it is necessary to carry out an extensive biogeochemical investigation in the Qingshui River. Multiple hydrochemical, isotopic (δ^2^H-H_2_O, δ^18^O-H_2_O and δ^13^C-DIC) and molecular-biological proxies are used to determine the biogeochemical co-formation processes of HCO_3_–Ca water and NO_3_^−^ pollution in piedmont agricultural areas.

## 2. Materials and Methods

### 2.1. Site Information and Sampling Campaign

**Figure 1:**
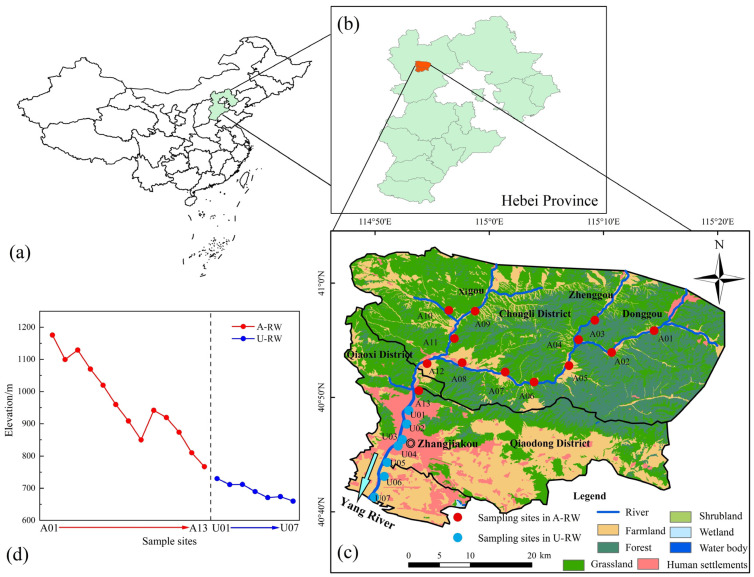
(**a**,**b**) Location of the Qingshui River in northwestern Hebei Province, China; (**c**) Land-use types and sampling sites in the upstream (A-RW) and downstream (U-RW) regions of the Qingshui River; (**d**) Elevation levels of the sampling sites.

The investigated area (40°39′ N–41°03′ N and 114°47′ E–115°20′ E) is located in the northwestern region of Hebei Province, China (Figure 1a–c). The area is characterized by a temperate continental monsoon and an arid/semi-arid climate, with average annual temperatures of 19 °C in summer and −12 °C in winter. The average annual evaporation is higher than the level of average annual precipitation. The elevation of sampling sites in the piedmont area in the northern region of the Qingshui River ranged from 786 to 1181 m, while in the basin area in the southern region, they ranged from 660 to 757 m (Figure 1d). The thin layer of quaternary sediment has been shown to be widely distributed, with carbonate rocks mainly distributed in the Lower Paleozoic system of the Qingshui River basin [34]. Water–rock interactions are facilitated by the large hydraulic slope and thin soil layer in the piedmont agricultural area.

The Qingshui River starts with the south part of Huapiling and consists of three tributaries (i.e., Donggou, Zhenggou and Xigou) in the mountain area, and rainwater is the main source of replenishment for the Qingshui River. After flowing through the A13 site, constrained by the mountains on both the east and west sides, and with the hardening of the riverbed, the river forms a concentrated flow from north to south in the urban area; then, it flows through Zhangjiakou City and eventually into the Yang River [28]. Field investigations showed that farmlands were mainly distributed near the banks of the upstream region (i.e., piedmont agricultural area), accounting for ~25% of the total study area. Due to a lack of supervision and regulatory measures coupled with the weak anti-seepage capacity of the natural riverbed, the upstream river water is vulnerable to agricultural inputs (e.g., synthetic fertilizers, domestic sewage and manure). In the downstream region (i.e., basin urban area), despite intensive urban activities in the surrounding areas, the construction of anti-seepage channels combined with strict environmental supervision provide effective protection for the river water ecosystem from urban pollution inputs.

To ensure that all of the main land-use types and altitudes were considered, river water samples were collected during a single field campaign conducted from 20 to 22 September 2023. The 20 sampling sites were selected within the Qingshui River, covering the main agricultural reaches of the piedmont area (A-RW; A01–A13) and the main urban reaches of the basin area (U-RW; U01–U07) (Figure 1c). Although the temporal variability of water quality hardening and NO_3_^−^ pollution in the Qingshui River was not determined by a single field campaign, this study selected a typical period, which was characterized by the intensive human activities and rapid hydrogeochemical evolution processes; the accumulated pollution and hydrogeochemical evolution outcomes from earlier periods can still be revealed by the obtained data in this manuscript. To avoid vertical effects and collect representative river water samples, samples were collected from the lower, middle and upper layers and then combined in equal proportions, with all samples collected in pre-cleaned, high-density polyethylene bottles. During field sampling, the pH, DO content, water temperature (T), ORP and TDS content were analyzed in situ using a portable multiparameter instrument (HQ40d, Hach, Loveland, CO, USA). Before processing, samples for the analysis of anions, cations and stable isotopes (δ^2^H-H_2_O, δ^18^O-H_2_O and δ^13^C-DIC) were stored at 4, 4 and −20 °C, respectively. After pretreatment, molecular-biological samples were immediately stored on dry ice and transported to Shanghai City (China).

### 2.2. Hydrochemical, Isotopic and Microbial Analysis

The analytical methods and testing institutions used for the analysis of Mg^2+^, Ca^2+^, Na^+^, K^+^, SO_4_^2−^, Cl^−^, HCO_3_^−^, F^−^, NO_3_^−^, NO_2_^−^, NH_4_^+^, δ^2^H-H_2_O, δ^18^O-H_2_O and δ^13^C-DIC in the samples are shown in Table S1. The analytical precision of hydrochemical parameters was guaranteed using the comparative analysis of standard materials, repeat sample analysis and reagent blanks. The charge balance error (CBE, %; Equation (1)) was employed to determine the reliability of the hydrochemical data, showing that the CBE values of all samples (0.64–3.16%) were below 5% and acceptable. International calibration standards and laboratory calibration standards were used to control the precision of δ^2^H, δ^18^O and δ^13^C analyses. The absolute errors of δ^2^H, δ^18^O and δ^13^C were better than ±1‰, ±0.2‰ and ±0.1‰, respectively. The obtained isotopic results were displayed as delta (δ, ‰; Equation (2)) and referred to the V-SMOW (δ^2^H-H_2_O and δ^18^O-H_2_O) and V-PDB (δ^13^C-DIC). The value of deuterium excess (*d*-excess) was calculated according to Equation (3) [35].

(1) $$\mathrm{CBE}=|\frac{\sum \;Anions-\sum Cations}{\sum \;Anions+\sum \;Cations}|\times 100\%$$

Δ = (*R_sample_*/*R_standard_* – 1) × 1000‰ (2)

*d*-excess = δ^2^H – 8δ^18^O (3)

The microbial composition of all samples was determined by, first, collecting material using a 0.22 μm sterile polycarbonate membrane and then using the FastDNA SPIN™ kit for soil (MP, Santa Ana, CA, USA) to extract DNA from the water samples. The obtained DNA concentrations were over 10 ng/μL, and the values of OD_260_/OD_230_ and OD_260_/OD_280_ were <2.0 and 1.8 ± 0.3, respectively, suggesting that the purification and concentrations of DNA were acceptable. Microbial communities were analyzed using high-throughput sequencing by Shanghai Majorbio Bio-pharm Technology Co., Ltd. (Shanghai, China). The 515F-907R primer pair was selected to amplify the V4–V5 hypervariable regions of bacterial 16S rRNA [36] using the GeneAmp 9700 thermocycler PCR system (ABI, Carlsbad, CA, USA). After collection and purification, the amplicons were pooled in equimolar and paired-end sequences (2 × 300) using the Illumina MiSeq platform (Illumina, San Diego, CA, USA).

### 2.3. Data Analysis

**Figure 2:**
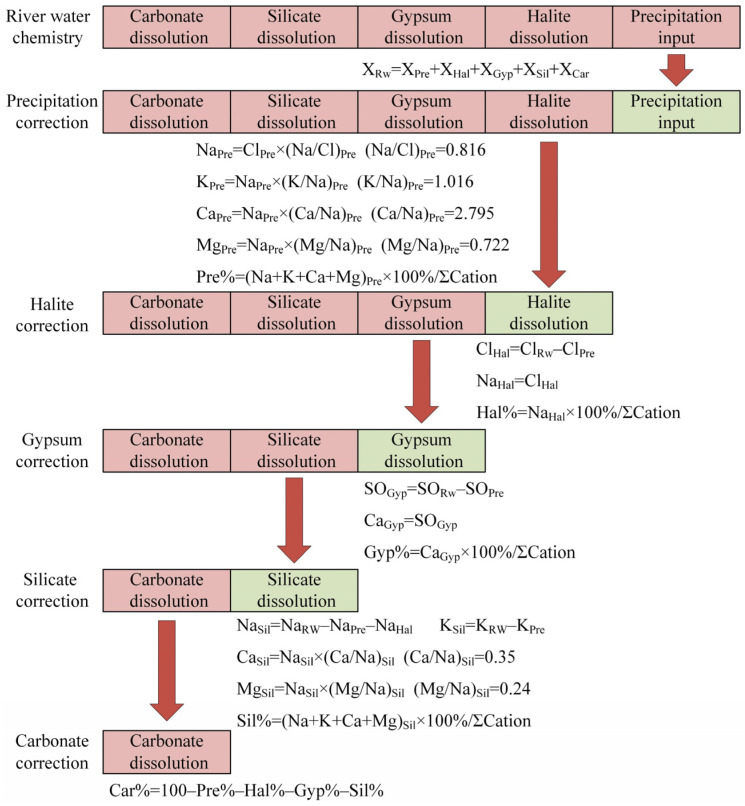
Flowchart of the forward model used to assess the proportional contributions of the five main sources to hydrochemical ions. Note: the values of (Ca/Na)_Sil_ and (Mg/Na)_Sil_ referred to Gaillardet et al. [37].

The FM (Figure 2) and SI were used together to evaluate the driving mechanisms of the riverine hydrochemistry. Bioinformatics assessment was used to elucidate nitrification function and its correlation with concentrations of Ca^2+^, Mg^2+^ and NO_3_^−^. The details of the above analyses are shown in Text S1. The statistical analysis of the hydrochemical and isotopic data was performed using SPSS software (v. 22.0, IBM, Armonk, NY, USA). Considering that some parameters were not normally distributed, Spearman coefficient and one-way ANOVA were used to determine the significant correlations and differences, respectively, among the hydrogeochemical compositions of A-RW and U-RW, with correlations and differences considered significant at a threshold of *p* < 0.05. The visualizations of physico-chemical and isotopic parameters were performed using Origin software (v. 2022).

## 3. Results

### 3.1. Overall Riverine Hydrochemistry

The measured physico-chemical and isotopic parameters are summarized in Table 1. The river water was neutral to slightly alkaline, with average Ph values of 8.48 ± 0.28 and 8.86 ± 0.25 for the A-RW and U-RW, respectively, suggesting alkaline mineral weathering. TDS values of all samples (415 ± 58.9 mg/L for A-RW and 267 ± 22.8 mg/L for U-RW) were within the WHO acceptable limit for drinking water (ALDW, 1000 mg/L) [38]. Due to the construction of anti-seepage channels and the growth of aquatic plants in the U-RW, the slight anaerobic condition promotes the rapid reduction in NO_3_^−^ in river water, and carbonate rocks no longer continue to dissolve. Hence, significantly higher concentrations of Ca^2+^ (90.9 ± 8.63 mg/L; *p* < 0.01), HCO_3_^−^ (285 ± 63.6 mg/L; *p* < 0.01) and NO_3_^−^ (35.6 ± 12.5 mg/L; *p* < 0.01) were detected in the A-RW compared to the U-RW, indicating that high TDS concentrations in the A-RW may be associated with carbonate dissolution and agricultural nitrogen emissions [30,31].

Piper diagrams are commonly used to evaluate the dominant ions in aquatic systems and hydrochemical facies [39]. HCO_3_^−^ and Ca^2+^ were the dominant anions and cations, respectively, with the main hydrochemical facies of HCO_3_–Ca in the A-RW (Figure S1) indicating that calcite dissolution was the main contributor to dissolved loads in the piedmont agricultural area. This result is similar to the findings of previous investigations conducted in other piedmont areas (e.g., the Hohhot Basin [40] and Po Plain [41]). However, the U-RW samples were closer to the mixed-type zone, with significantly higher concentrations of Cl^−^ (43.2 ± 3.32 mg/L; *p* < 0.05). Riverine Cl^−^ concentrations in the U-RW were significantly correlated with Mg^2+^ (*R* = 0.86; *p* < 0.05) and showed a low level of correlation with Na^+^ (*R* = 0.68), HCO_3_^−^ (*R* = 0.61) and NH_4_^+^ (*R* = 0.65; Figure S2), reflecting the combined effects of Cl-containing mineral dissolution and anthropogenic emissions.

### 3.2. Isotopic Parameters

The isotopic values of δ^2^H-H_2_O, δ^18^O-H_2_O and δ^13^C-DIC ranged from −80.0 to −43.0‰, −10.8 to −4.50‰ and −12.0 to −3.88‰, respectively (Table 1). The isotopic values of the A-RW samples (−73.2 ± 5.18‰ for δ^2^H-H_2_O, −9.09 ± 0.84‰ for δ^18^O-H_2_O and −10.6 ± 1.14‰ for δ^13^C-DIC) exhibited significantly lower levels (*p* < 0.05) compared to those of the U-RW samples (−53.3 ± 7.89‰ for δ^2^H-H_2_O, −6.66 ± 1.41‰ for δ^18^O-H_2_O and −5.01 ± 1.08‰ for δ^13^C-DIC).

### 3.3. Microbial Communities

**Figure 3:**
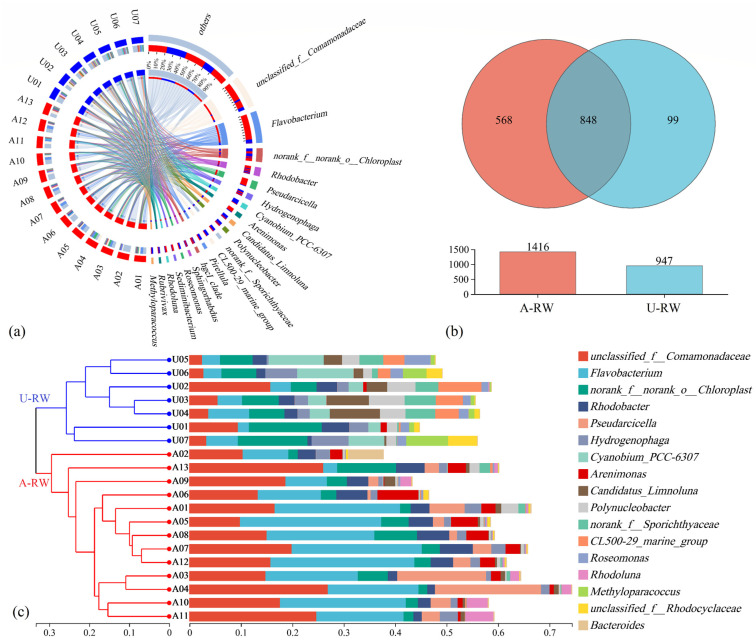
Microbial communities at the genus level in river water in the upstream (A-RW) and downstream (U-RW) regions of the Qingshui River (China). (**a**) Taxonomic composition and relative abundance; (**b**) Core and unique genera between A-RW and U-RW regions; (**c**) Spatial distribution patterns with hierarchical clustering across sampling sites.

As shown in Figure 3a, the dominant genera in the study area were *unclassified f Comamonadaceae*, *Flavobacterium* and *norank f norank o Chloroplast*, with high relative abundances of 13.6%, 13.4% and 5.71%, followed by *Rhodobacter* (3.62%) and *Pseudarcicella* (3.42%). Differences were observed in the microbial community structures along the river flow pathway, with 568 unique genera detected in the A-RW and 99 unique genera detected in the U-RW (Figure 3b), indicating that the A-RW contained a higher abundance of functional microbes and a more diverse microbial community. Furthermore, the microbes within the community were classified into two groups by hierarchical clustering based on Bray Curtis analysis (i.e., A-RW: A01-A13 and U-RW: U01-U07; Figure 3c), which were consistent with the different types of land-use and topography.

## 4. Discussion

### 4.1. Environmental Factors

#### 4.1.1. Evaporation and Recharge Processes

The contribution of evaporation to water hardening and hydrochemical evolution cannot be ignored in arid/semi-arid areas [19,42]. As shown in Figure S3a, the δ^18^O-H_2_O/δ^2^H-H_2_O values of all samples fell below the global meteoric water line (GMWL, δ^2^H-H_2_O = 8δ^18^O-H_2_O + 10) [43], indicating an overall trend of evaporation. The post-condensation evaporative effect is likely to occur in the A-RW due to the values of *d*-excess varying from 0 to 10‰ (Figure S3b) [44,45]. The arid/semi-arid climate coupled with the evaporation process facilitates the formation of alkaline water conditions [46], which may be associated with the dissolution of alkaline minerals (e.g., calcite and dolomite minerals). Based on the obtained observations, it may be proposed that the Qingshui River is recharged via at least three main processes: (1) run-off from mountainous areas, (2) atmospheric precipitation and (3) irrigation water and domestic sewage (Figure S3c), as determined by the wide range of Cl^−^ concentrations (corresponding to irrigation return flow and domestic sewage). Agricultural emissions contain large amounts of nitrogen-containing pollutants, especially NH_4_^+^ [9,32,45], which may be a crucial factor contributing to riverine NO_3_^−^ pollution in the A-RW.

#### 4.1.2. Topography and Channel Structure

The spatial variability of water facies is controlled, to some extent, by the topography and channel structure of the study area. The topographical features of the piedmont area have characteristics of a high topographic slope and fast water flow (Figure 1d), accelerating water–rock interaction processes and enhancing the solubility of minerals (e.g., halite and gypsum), causing them to preferentially dissolve into river water. Over a long-term period, water–rock interactions result in a gradual decrease in the availability of easily soluble minerals. The dissolution of the remaining less-soluble minerals (e.g., dolomite and calcite) is a primary factor controlling hydrochemical evolution, ultimately resulting in HCO_3_–Ca water in the A-RW (Figure S1) [33]. In contrast, the U-RW is characterized as a smaller hydraulic slope (Figure S1) that, coupled with the construction of rubber dams and anti-seepage channels, significantly slows down the river water flow and hinders natural carbonate dissolution. Hence, the water facies in the U-RW tends to be mixed-type (Figure S1). In addition, natural river beds have been shown to have a limited capacity to block agricultural pollutants due to the widespread and abundant occurrence of spaces among the loose deposits. Thus, river water ecosystems are particularly sensitive to agricultural nitrogen emissions [47]. In contrast, although the number of pollutants originating from urban activities are higher than those originating from agricultural activities, the construction of anti-seepage channels prevents urban sewage from flowing into the river to some extent, and with the inclusion of strict environmental supervision measures, river water quality is somewhat less affected by urban pollutants.

### 4.2. Mineral Dissolution Dominated by Carbonate Rocks

#### 4.2.1. Hydrochemical Indicators

**Figure 4:**
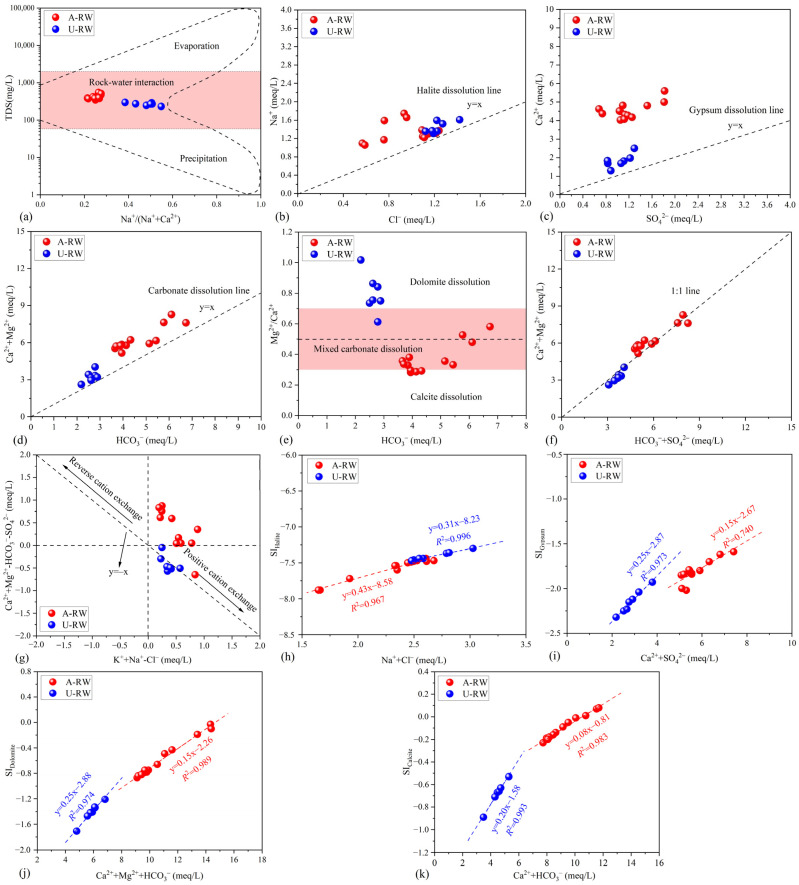
(**a**–**g**) Hydrochemical indicators and (**h**–**k**) saturation index illustrating the mineral dissolution dominated by carbonate rocks.

The Gibbs model is an effective tool for evaluating the main mechanism controlling world surface water chemistry (i.e., evaporation, rock–water interaction and precipitation) [48]. In this study, water hardening was found to be mainly controlled by rock–water interactions, while precipitation and evaporation were not the primary natural mechanisms driving riverine hydrochemistry (Figure 4a). The TDS values in the A-RW (415 ± 58.9 mg/L) were 1.55-fold higher than in the U-RW (267 ± 22.8 mg/L; Table 1), suggesting a stronger role of water–rock interactions in the A-RW. Furthermore, the neutral to slightly alkaline water environment coupled with HCO_3_–Ca water being the dominant hydrochemical type may be related to carbonate dissolution (Table 1 and Figure S1) [31]. The TDS content was positively correlated with Mg^2+^, Ca^2+^ and HCO_3_^−^ in both the A-RW and U-RW (Figure S2), providing further support for carbonate dissolution being the main factor controlling hydrochemical evolution.

In theory, if riverine Cl^−^ and Na^+^ originate only from halite dissolution, the milliequivalents of Na^+^ and Cl^−^ in water should be equivalent (Equation (4)). When the ratio of Na^+^ and Cl^−^ is >1 or <1, the hydrochemistry may be influenced by silicate dissolution or cation exchange, respectively [49]. The results showed that the Na^+^:Cl ratios of all samples were along the upper left portion of the halite dissolution line (i.e., Na^+^:Cl^−^ = 1:1; Figure 4b), which may be attributed to the dissolution of silicate. Similarly, gypsum dissolution theoretically releases equal milliequivalents of Ca^2+^ and SO_4_^2−^ into river water (Equation (5)). The milliequivalent of Ca^2+^ exceeded that of SO_4_^2−^, with all samples falling into the excess zone of Ca^2+^ (Figure 4c), indicating additional Ca-containing minerals were responsible for the Ca^2+^ excess (such as carbonate) [24,25]. Carbonate dissolution by H_2_CO_3_ results in a 1:1 ratio of Ca^2+^ + Mg^2+^ and HCO_3_^−^ [18]. All samples were slightly above the carbonate dissolution line (Figure 4d) and were closer to the y = x line relative to gypsum dissolution (Figure 4c), implying that carbonate dissolution had an intensive influence on hydrochemical formation. Furthermore, the dissolution of dolomite and calcite releases Ca^2+^ and Mg^2+^ into river water with ratios of 1:1 and 1:0, respectively (Equations (6) and (7)) [50]. Most of the A-RW samples fell within the mixed-carbonate dissolution zone and tended towards the calcite dissolution zone (Figure 4e), indicating that water hardening was mainly controlled by calcite dissolution, with a small contribution from dolomite dissolution.

Positive cation exchange is beneficial, as it alleviates water hardening but increases water salinity [46]. When positive cation exchange occurs (i.e., the desorption of Na^+^ and K^+^ coupled with the adsorption of Mg^2+^ and Ca^2+^, Equation (8)) [19], the milliequivalent of HCO_3_^−^ + SO_4_^2−^ is higher than that of Ca^2+^ + Mg^2+^ with an excess of anions [51]. In this study, cation exchange may provide a low contribution to riverine hydrochemistry, as almost all samples were close to the 1:1 line (Figure 4f). To further determine the importance and role of cation exchange, the plot of (Na^+^ + K^+^ − Cl^−^) versus (Mg^2+^ + Ca^2+^ − HCO_3_^−^ − SO_4_^2−^) is shown in Figure 4g [52]. Most U-RW samples were located along the *y* = −*x* line, reflecting a trend of positive cation exchange. However, the A-RW samples were located far from the 0 end-member and did not fit on the *y* = −*x* line, suggesting that positive cation exchange had a weak capacity to alleviate water hardening. Similar results have been reported for other piedmont areas in the Tarim Basin, China [46], and the Himalayan foothills river basin, India [53].

NaCl→Cl^−^ + Na^+^ (4)

CaSO_4_⋅2H_2_O→SO_4_^2–^ + Ca^2+^ + 2H_2_O (5)

2H_2_O + 2CO_2_ + CaMg(CO_3_)_2_→Mg^2+^ + Ca^2+^ + 4HCO_3_^−^ (6)

H_2_O + CO_2_ + CaCO_3_→Ca^2+^ + 2HCO_3_^−^ (7)

(Na^+^ + K^+^)_mineral_ + (Mg^2+^ + Ca^2+^)_water_→(Mg^2+^ + Ca^2+^)_mineral_ + (Na^+^ + K^+^)_water_ (8)

#### 4.2.2. Saturation Index

Determination of the dissolution equilibrium of minerals is imperative to understanding water hardening and hydrogeochemical processes [20,54]. Positive linear regression relationships were determined between ion concentrations and the corresponding SI (Figure 4h–k). The values of SI_Halite_, SI_Gypsum_, SI_Dolomite_ and SI_Calcite_ exhibited good linear relationships with the corresponding hydrochemical compositions (*R*^2^ = 0.740–0.996), indicating the main contribution of mineral dissolution to riverine hydrochemistry. The dissolution of halite (SI_Halite_ = −7.58 ± 0.15 < 0), gypsum (SI_Gypsum_ = −1.81 ± 0.12 < 0) and dolomite (SI_Dolomite_ = −0.58 ± 0.30 < 0) in the A-RW was in an undersaturated state, while the SI_Calcite_ value (23.1%) in the A-RW samples exceeded 0 slightly, indicating an equilibrium/supersaturated state. In contrast, the SI values of all U-RW samples were <0, confirming the undersaturated state. The SI_Halite_ values in the U-RW samples were higher than in the A-RW samples, and the higher contribution of halite dissolution may be associated with the positive cation exchange trend in the basin area [52]. The SI_Calcite_, SI_Dolomite_ and SI_Gypsum_ values in the A-RW were higher than those in the U-RW, inferring that the combination of rapid hydrodynamic conditions with natural river beds can accelerate rock–water interaction processes. Furthermore, the equilibrium/supersaturated state of calcite dissolution may be responsible for HCO_3_–Ca water in the A-RW. In addition to natural carbonate dissolution, the contribution of agricultural acid (i.e., HNO_3_) to the equilibrium/supersaturated state of calcite dissolution may be significant due to the large agricultural NH_4_^+^ inputs in the study area [28], which can accelerate the rate of dissolution from carbonate rocks, thereby improving the equilibrium/supersaturated state of calcite dissolution.

#### 4.2.3. Forward Model

**Figure 5:**
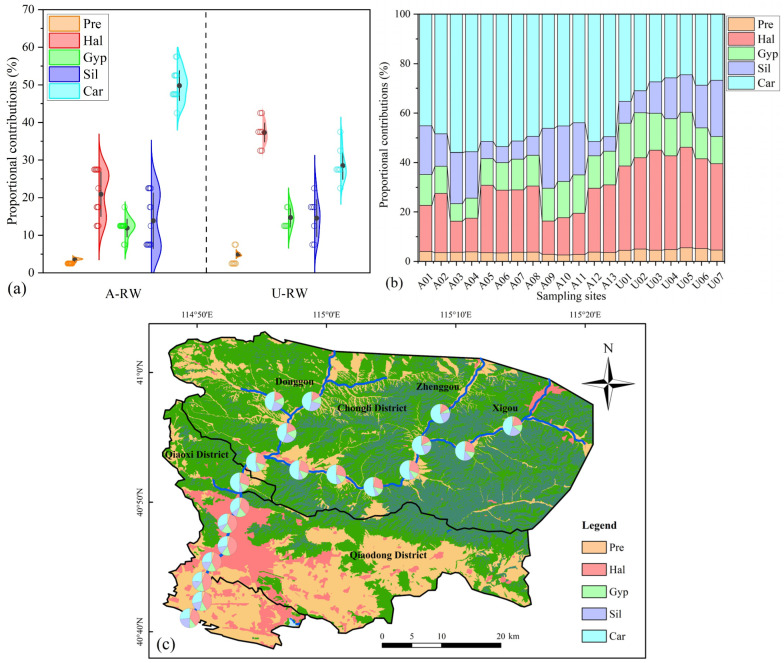
Proportional contributions of precipitation input (Pre), halite dissolution (Hal), gypsum dissolution (Gyp), silicate dissolution (Sil) and carbonate dissolution (Car) to the hydrochemical composition of the Qingshui River. (**a**) FM-estimated fractions; (**b**) site-specific contributions; (**c**) spatial distribution.

According to the FM outputs (Figure 5a), the percentage contributions of natural hydrochemical sources were ranked as Car (49.8 ± 3.96%) > Hal (20.9 ± 5.94%) > Sil (13.9 ± 7.40%) > Gyp (11.9 ± 2.39%) > Pre (3.49 ± 0.43%) for the A-RW and Hal (37.3 ± 2.51%) > Car (28.5 ± 3.64%) > Gyp (14.7 ± 2.52%) > Sil (14.6 ± 4.94%) > Pre (4.89 ± 0.39%) for the A-RW. Thus, carbonate dissolution was determined to be the primary contributor to riverine hydrochemistry in the piedmont agricultural study area. Furthermore, HCO_3_–Ca water was mainly driven by calcite dissolution according to the stoichiometric relationship (Equations (6) and (7)) and equilibrium/supersaturated state of calcite dissolution (Figure 4e,k). The relative contribution of Pre was the lowest in all samples at 3.98 ± 0.80%, which is similar to the reported findings from other arid/semi-arid areas (e.g., Eastern Tibet; Yellow River Basin) [20,55]. The relative contribution of Sil and Gyp were similar in the samples from both the A-RW and U-RW, whereas those of Car and Hal exhibited significant differences (*p* < 0.05) in spatial distribution between the A-RW and U-RW (Figure 5b,c). For the basin area (U-RW), riverine hydrochemistry may be directly affected by the upstream residual river sediments and artificial channel materials. Furthermore, positive cation exchange promoted the adsorption of divalent ions (Ca^2+^ and Mg^2+^) and the desorption of monovalent ions (Na^+^ and K^+^) under alkaline conditions (Figure 4g) [46]. However, the absence of positive cation exchange coupled with carbonate dissolution providing the largest contribution resulted in the prevalence of HCO_3_–Ca water in the A-RW. Overall, the HCO_3_–Ca water was significantly controlled by carbonate (especially calcite) dissolution in the A-RW.

### 4.3. Agricultural NH_4_^+^ Emissions

**Figure 6:**
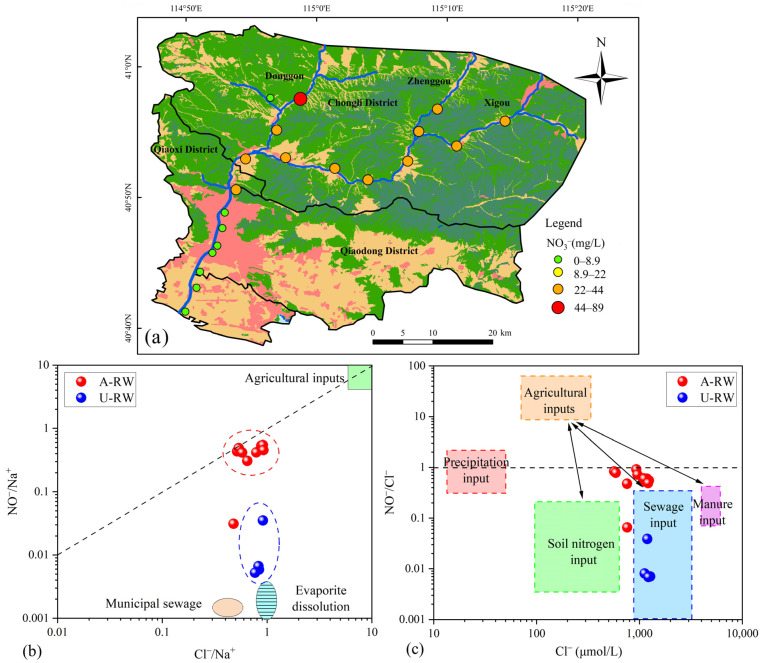
Spatial variability of NO_3_^−^ concentrations in the upstream (A-RW) and the downstream (U-RW) regions of the Qingshui River; (**a**) Spatial distribution map of NO_3_^−^ concentration; (**b**) Diagram showing the molar ratios of Cl^−^/Na^+^ versus NO_3_^−^/Na^+^ in A-RW and U-RW river water samples; (**c**) Variations in Cl^−^ concentration (μmol/L) vs. NO_3_^−^/Cl^−^ (molar ratio) (adapted from Torres-Martínez et al.) [45].

Agricultural NH_4_^+^-containing pollutants (e.g., NH_4_HCO_3_, NH_4_Cl, manure, sewage and soil organic nitrogen) are the main sources of NO_3_^−^ in both river water and groundwater around the world (Table 2). The results showed that NO_3_^−^ concentrations in the A-RW, where agricultural activities were relatively intensive, were ~31.8-fold higher than those in the U-RW, reflecting the effects of agricultural NH_4_^+^ emissions on nitrogen pollution in the A-RW (Figure 6a). To further determine the influence of agricultural NH_4_^+^ emissions on riverine pollution, a scatter plot was prepared for Cl^−^/Na^+^ versus NO_3_^−^/Na^+^, as shown in Figure 6b. Most of the A-RW samples were located close to the *y* = *x* line in the end-member region for agricultural inputs, indicating that riverine hydrochemistry was controlled, to some extent, by agricultural inputs. However, the A-RW samples did not fall into any specific NO_3_^−^ source zones (Figure 6c), indicating that this region was affected by a mixture of NH_4_^+^ inputs, including manure, sewage and soil nitrogen sources [45]. Based on a previously reported investigation in the A-RW in 2022, the total contribution rates of agricultural NH_4_^+^ emissions (i.e., manure, domestic sewage, soil nitrogen and NH_4_^+^-synthetic fertilizer) to the riverine NO_3_^−^ content reached up to 99.4% [28], and no significant changes were observed in the agricultural production practices or local community characteristics between the two study periods in the A-RW. Indeed, although agricultural land (rural settlements and farmland) accounted for only ~16% of the upstream river area, farmlands and villages were located mainly on both sides of the riverbank. NH_4_^+^-synthetic fertilizers, such as NH_4_HCO_3_ and CO(NH_2_)_2_, are commonly applied excessively in order to promote the growth of greenhouse crops (such as strawberries, mushrooms, tomatoes and cucumbers). Manure is added widely to farmland crops to provide nutrients, with the domestic sewage and livestock waste used as manure not generally well treated due to the limitations of local sewage treatment systems. All of these aspects increase the volume of agricultural NH_4_^+^ emissions, causing further deterioration of river water quality. Therefore, it can be inferred that these agricultural NH_4_^+^ emissions are crucial contributors to riverine NO_3_^−^ pollution within the study area.

### 4.4. Biogeochemical Mechanisms

Microbial nitrification is a common pathway in natural oxygen-rich aquatic ecosystems [45,59], oxidizing NH_4_^+^ to NO_3_^−^. The combination of rapid hydrodynamic conditions and aerobic water environments in piedmont areas facilitates microbial nitrification [22,60]. Notably, HNO_3_ (i.e., H^+^+NO_3_^−^; Equation (9)) can be produced by nitrifying bacteria from the oxidized form of agricultural NH_4_^+^ [32]. In the A-RW, high NO_3_^−^ concentrations (3.08–52.8 mg/L) accompanied by low concentrations of NO_2_^−^ (below the detection limit; BDL) and NH_4_^+^ (BDL-0.16 mg/L; Table 1) may be attributed to the occurrence of microbial nitrification [13]. Furthermore, a combination of high DO levels (10.1 ± 1.45 mg/L) with neutral to slightly alkaline pH conditions (8.48 ± 0.28) is suitable for the metabolism of nitrifying bacteria [45]. When NH_4_^+^ is oxidized to HNO_3_ through nitrification processes, 2/3 O-NO_3_^−^ is theoretically derived from ambient water, with the remaining O-NO_3_^−^ derived from atmospheric oxygen (Equation (10)) [26,27]. Based on the results of isotopic kinetic fractionation, the nitrification processes preferentially utilize lighter isotopes (δ^16^O-H_2_O), making δ^18^O-H_2_O more likely to accumulate in the river water. The obtained δ^18^O-H_2_O values (−9.09 ± 0.84‰; Table 1) in the A-RW were close to the reference values reported for the effect of nitrification processes (−10.0 ± 0.83‰) [28], indicating that microbial nitrification plays a major role in the A-RW, while the U-RW values (−6.66 ± 1.41‰) differed from the reference values, indicating that microbial nitrification played a smaller role.

**Figure 7:**
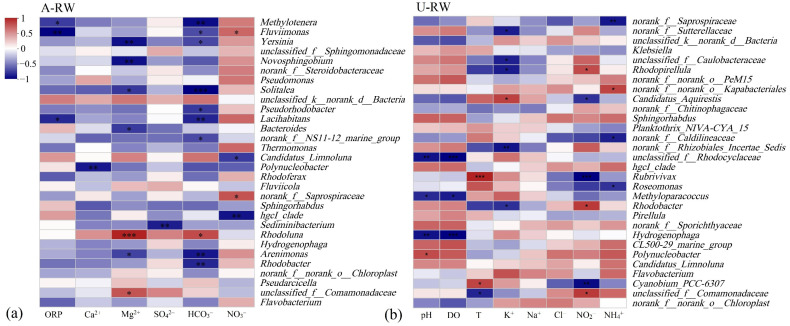

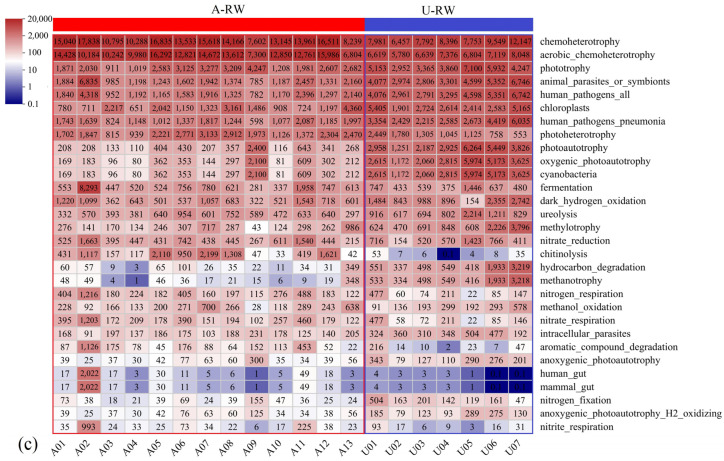
(**a**,**b**) Correlations between microbial compositions (at the genus level) and key physico-chemical factors and (**c**) frequencies of functional microbial communities in the upstream (A-RW) and downstream (U-RW) regions of the Qingshui River (China). Note: * for *p* < 0.05, ** for *p* < 0.01, and *** for *p* < 0.001.

According to the RDA outputs (Figure S4), riverine NO_3_^−^ was a key hydrochemical factor driving microbial community evolution in the A-RW, although it was not a main factor in the U-RW (Figure 7a,b). In the A-RW, riverine NO_3_^−^ was positively correlated with *Fluviimonas* (*p* < 0.05), *norank f Saprospiraceae* (*p* < 0.05), *Flavobacterium*, *Rhodobacter* and *Arenimonas* (Figure 7a), which may be associated with nitrification processes. The relative abundance of the dominant genus *Flavobacterium* (Figure 3a) was 3.96-fold higher in the A-RW (18.1 ± 8.09%) than in the U-RW (4.57 ± 1.93%), while the total relative abundance of all genera associated with riverine NO_3_^−^ was close to 21% in the A-RW. This indicates that microbial nitrification had a major advantage during ammonia oxidation processes in the piedmont agricultural area. The observed frequencies of functional microbial communities provided further support for HNO_3_ being dominantly derived from ammonia oxidation via nitrification (Figure 7c). The frequencies of chemoheterotrophs and aerobic chemoheterotrophs in the A-RW (12,149 ± 3044 and 13,283 ± 3242, respectively) were significantly higher than those in the U-RW (8582 ± 1820 and 6998 ± 770, respectively; *p* < 0.01). Although the chemoautotrophic nitrifying bacteria have faster NH_4_^+^ oxidation rates, chemoheterotrophic nitrifying bacteria are more tolerant to complex aquatic ecosystems, which may contribute more to HNO_3_ in the A-RW (Figure 7c). Additionally, microbial communities may be co-controlled by Ca^2+^, Mg^2+^, HCO_3_^−^ and SO_4_^2−^ in the A-RW, to a certain extent (Figure S4). Only *unclassified k norank d Bacteria* (<0.05%) and *Rhodoluna* (1.32 ± 1.71%) were positively correlated with Ca^2+^, Mg^2+^, HCO_3_^−^ and SO_4_^2−^ (Figure 7a), possibly due to the production of H_2_SO_4_ by sulfur oxidation, which is subsequently involved in carbonate dissolution. However, *Rhodoluna* had a relatively high abundance in sites A11 and A12 (4.05 and 5.41%, respectively), corresponding to higher SO_4_^2−^ concentrations (87.2 and 86.9 mg/L, respectively), while it contributed less to SO_4_^2−^ concentrations in other sampling sites. Overall, microbial nitrification processes occurred widely in the A-RW of the Qingshui River, further increasing the amounts of H^+^ and NO_3_^−^ ions originating from the oxidization of agricultural NH_4_^+^-containing pollutants.

**Figure 8:**
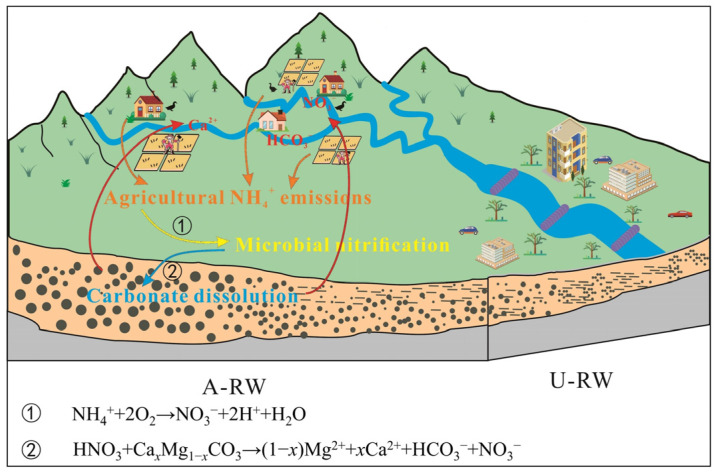
Conceptual model of the co-controlling effects of carbonate dissolution and agricultural NH_4_^+^ emissions on HCO_3_–Ca and NO_3_^−^ pollution in river water in the piedmont agricultural area.

In natural environments, carbonate is mainly dissolved by H_2_CO_3_ (Equations (6) and (7)). In this process, half of the dissolved inorganic carbon (DIC) originates from carbonate rocks, while the remaining DIC originates from atmospheric CO_2_ [22,24]. However, the involvement of HNO_3_ in carbonate dissolution does not require atmospheric CO_2_ (Equation (11)), causing some significant differences in the carbon isotopic fingerprints and hydrochemical signatures of samples [61]. The δ^13^C-DIC values from CO_2_ in karst regions have been reported to range from −20.4 to −9.3‰ after isotopic fractionation, while carbonate exhibits enriched levels of δ^13^C-DIC (~0‰) [31,62]. In the present study, the lowest δ^13^C-DIC value was −12‰, and the corresponding equivalent (Ca^2+^ + Mg^2+^)/HCO_3_^−^ ratio was 1.13, which is close to the theoretical natural carbonate dissolution value ((Ca^2+^ + Mg^2+^)/HCO_3_^−^ = 1; Figure S5a). If carbonate is only dissolved by strong acids, the δ^13^C-DIC value would theoretically be equal to 0‰, and the equivalent ratio of (Ca^2+^ + Mg^2+^)/HCO_3_^−^ would be 2 [31]. In the present study, all samples were distributed between the end-members of carbonate dissolution by H_2_CO_3_, HNO_3_ and H_2_SO_4_, indicating the involvement of anthropogenic acids in natural carbonate dissolution. The diagram of SO_4_^2−^/HCO_3_^−^ vs. (Ca^2+^ + Mg^2+^)/HCO_3_^−^ provides further understanding of the mechanism of carbonate rock dissolution (Figure S5b). All samples tended to be located towards the end-members of carbonate dissolution by H_2_CO_3_ and HNO_3_, whereas most of the samples deviated from the end-member of carbonate dissolution by H_2_SO_4_, indicating that carbonate was mainly dissolved by H_2_CO_3_, followed by HNO_3_, while H_2_SO_4_ contributed less to carbonate dissolution (especially calcite). These results are consistent with the results of microbial analysis (Figure 7). Overall, in the study area, a large amount of agricultural NH_4_^+^ emissions were converted into HNO_3_ via microbial nitrification, which accelerated carbonate dissolution and promoted the rapid release of Ca^2+^, Mg^2+^, HCO_3_^−^ and NO_3_^−^ into river water in the A-RW area (Figure 8). These biogeochemical processes altered the natural mechanisms of riverine hydrochemistry, aggravating water hardening and NO_3_^−^ pollution in river water in the piedmont agricultural area. Therefore, the biogeochemical mechanisms should be considered in future hydrochemical investigations and water environment management in other piedmont agricultural areas around the world.

2O_2_ + NH_4_^+^→H_2_O + NO_3_^−^ + 2H^+^ (9)

δ^18^O-NO_3_^−^ = 2/3δ^18^O-H_2_O + 1/3δ^18^O-O_2_ (10)

HNO_3_ + Ca*_x_*Mg_1−*x*_CO_3_→(1 − *x*)Mg^2+^ + *x*Ca^2+^ + HCO_3_^−^ + NO_3_^−^ (11)

## 5. Conclusions

This study reports the combined use of multiple hydrochemical, isotopic and molecular-biological proxies along with the FM and SI to establish the co-driving mechanisms of riverine HCO_3_–Ca water and NO_3_^−^ pollution in a typical piedmont agricultural area. Riverine HCO_3_–Ca water and NO_3_^−^ pollution (up to 52.8 mg/L) were widely detected in the A-RW. The primary contributor of mineral dissolution to riverine hydrochemistry was Car (49.8 ± 3.96%), followed by Hal (20.9 ± 5.94%), Sil (13.9 ± 7.40%) and Gyp (11.9 ± 2.39%), whereas Pre contributed less to riverine hydrochemistry in the A-RW (3.49 ± 0.43%). Water hardening was mainly driven by carbonate dissolution, particularly calcite. Riverine NO_3_^−^ was derived from agricultural NH_4_^+^ emissions (e.g., manure, domestic sewage, soil nitrogen and NH_4_^+^-synthetic fertilizers). The rapid hydrodynamic conditions and aerobic water environment in the piedmont area were conducive to the metabolism of nitrifying bacteria, which promoted the production of HNO_3_ from agricultural NH_4_^+^ via microbial nitrification (e.g., *Flavobacterium* and *Fluviimonas*). The produced HNO_3_ was preferentially involved in carbonate dissolution, which was attributed to the strong acidity of HNO_3_, resulting in the release of Ca^2+^, Mg^2+^, HCO_3_^−^ and NO_3_^−^ into river water. These biogeochemical mechanisms could be responsible for riverine HCO_3_–Ca and NO_3_^−^ pollution in the piedmont agricultural area. However, the co-enrichment mechanisms of Ca^2+^, Mg^2+^, HCO_3_^−^ and NO_3_^−^ were not prevalent in the U-RW due to the limited role of agricultural NH_4_^+^ emissions and insufficient carbonate dissolution in this region. Therefore, the implementation of strict control measures for agricultural NH_4_^+^ emissions is a fundamental aspect of the alleviation of water hardening and NO_3_^−^ pollution, such as the use of slow-release NH_4_^+^-synthetic fertilizers, the application of nitrogen fertilizer synergists, the precise fertilization of root soils and the establishment of scientific fertilization plans based on the growth characteristics of plants. This study highlights the significant influences of carbonate dissolution and agricultural NH_4_^+^ emissions on riverine water quality and provides an example of the biogeochemical mechanisms driving riverine HCO_3_–Ca water and NO_3_^−^ pollution, which may be relevant for other piedmont agricultural areas.

## Figures and Tables

**Table 1:** Statistical summary of descriptive statistics for hydrochemical and isotopic data in the upstream (A-RW) and downstream (U-RW) regions of the Qingshui River.

Parameters	Unit	A-RW (*n* = 13)					U-RW (*n* = 7)				
		Mean	Min.	Max.	SD	CV	Mean	Min.	Max.	SD	CV
pH	/	8.48	7.75	8.75	0.28	0.03	8.86	8.46	9.18	0.25	0.03
DO	mg/L	10.1	8.40	13.7	1.45	0.14	10.6	3.79	15.0	4.26	0.40
T	°C	15.0	12.1	18.4	2.20	0.15	20.0	18.0	22.2	1.37	0.07
ORP	mV	−76.4	−92.7	−33.7	16.0	0.21	−100	−123	−75.2	17.4	0.17
TDS	mg/L	415	346	546	58.9	0.14	267	230	296	22.8	0.09
K^+^	mg/L	3.33	0.37	5.07	1.38	0.41	5.86	4.30	7.72	1.22	0.21
Na^+^	mg/L	30.9	24.3	40.1	4.81	0.16	33.2	30.1	37.0	2.87	0.09
Ca^2+^	mg/L	90.9	80.6	112	8.63	0.09	36.5	25.9	50.0	7.30	0.20
Mg^2+^	mg/L	20.6	13.6	33.5	6.93	0.34	17.0	15.3	18.4	1.21	0.07
Cl^−^	mg/L	34.0	19.9	43.4	8.18	0.24	43.2	39.3	49.7	3.32	0.08
SO_4_^2−^	mg/L	57.1	32.7	87.3	16.6	0.29	49.6	39.7	62.1	9.06	0.18
HCO_3_^−^	mg/L	285	223	411	63.6	0.22	160	134	176	14.2	0.09
NO_3_^−^	mg/L	35.6	3.08	52.8	12.5	0.35	1.12	0.52	2.86	1.16	1.03
NO_2_^−^	mg/L	/	BDL	BDL	/	/	1.38	1.03	1.69	0.35	0.26
NH_4_^+^	mg/L	/	BDL	0.16	/	/	0.35	0.24	0.48	0.07	0.21
F^−^	mg/L	/	BDL	0.03	/	/	/	BDL	BDL	/	/
δ^2^H-H_2_O	‰	−73.2	−80.0	−63.0	5.18	0.07	−53.3	−66.0	−43.0	7.89	0.15
δ^18^O-H_2_O	‰	−9.90	−10.8	−8.20	0.84	0.08	−6.66	−8.70	−4.50	1.41	0.21
*d*-excess	‰	6.05	2.60	7.80	1.74	0.29	−0.03	−7.00	3.60	3.62	/
δ^13^C-DIC	‰	−10.6	−12.0	−8.60	1.14	0.11	−5.01	−7.12	−3.88	1.08	0.22

**Table 2:** Statistical results on the contribution of NH_4_^+^-containing pollutants to NO_3_^−^ in water bodies.

Regions	NH_4_^+^-Containing Pollutants	References
Comarca Lagunera, Mexico	Manure from concentrated animal-feeding operations (~48%), urban sewage (~43%), soil organic nitrogen (~4%), NH_4_^+^-synthetic fertilizers (~3%) and atmospheric deposition (~1%).	[45]
Huixian karst wetland, China	Atmospheric nitrogen deposition (3.44%), synthetic NH_4_^+^ fertilizer (36.6%), soil organic nitrogen (28.0%), domestic sewage and manure (15.1%).	[25]
Hohhot Basin’s Piedmont, China	Manure (20.5%), soil nitrogen (63.8%) and ammonia fertilizer (28.8%).	[40]
Nyando tropical river basin, Kenya	Ammonium in fertilizer/rain (10%), soil nitrogen (18–41%), manure and sewage (46–70%).	[56]
Bukit Merah Reservoir, Malaysia	Atmospheric deposition (23–29%), soil nitrogen (25–26%), manure and sewage (25–33%),	[57]
Sardinia, Italy	NH_4_^+^ fertilizers (2.13%), soil organic nitrogen (0.55%), sewage and manure (58.5%).	[58]

## Data Availability

The data that support the findings of this study are available from the corresponding author upon reasonable request.

## Supplementary Materials

The following supporting information can be downloaded at: https://www.mdpi.com/article/10.3390/toxics13050394/s1, Table S1: Analytical methods of chemical parameters and stable isotopes in river water samples in the study area; Table S2: Statistical summary of descriptive statistics for hydrochemical and isotopic data in the upstream (A-RW) and downstream (U-RW) regions of the Qingshui River. Note: d-excess = δ^2^H–8δ^18^O [35]; Max.: maximum; Min.: minimum; SD: standard deviation; CV: coefficient of variation; BDL: below detection limit;/: no calculated value; Figure S1: Piper diagram showing the hydrochemical facies, dominant cations and dominant anions of river water in the upstream (A-RW) and downstream (U-RW) regions of the Qingshui River; Figure S2: Correlation analysis of physico-chemical data in the upstream (A-RW; upper triangle) and the downstream (U-RW; lower triangle) regions of the Qingshui River; Figure S3: Diagrams of (a) δ^18^O-H_2_O versus δ^2^H-H_2_O, (b) TDS versus *d*-excess and (c) δ^18^O-H_2_O versus Cl^−^ in the upstream (A-RW) and the downstream (U-RW) of the Qingshui River. Note: the global meteoric water line (GMWL) referred to [43]; Figure S4: RDA analysis illustrating the effects of physico-chemical factors on microbial community structure; Figure S5: Involvement of strong acids in carbonate dissolution interpreted by scatter plot of (a) (Ca^2+^ + Mg^2+^)/HCO_3_^−^ versus δ^13^C-DIC (adapted from [31]) and (b) SO_4_^2−^/HCO_3_^−^ versus (Ca^2+^ + Mg^2+^)/HCO_3_^−^ (adapted from [23]) in the upstream (A-RW) and downstream (U-RW) regions of the Qingshui River; Text S1: Analysis on the joint use of FM and SI to evaluate the driving mechanism of river hydrochemistry [63,64].
