# Supplementary material for: Biogeochemical Mechanisms of HCO3–Ca Water and NO3− Pollution in a Typical Piedmont Agricultural Area: Insights from Nitrification and Carbonate Weathering
Source: Toxics. 2025 May 15;13(5):394. doi: 10.3390/toxics13050394 (PMC12115938; doi:10.3390/toxics13050394)
Supplement: Supplementary file 1 [file toxics-13-00394-s001.zip › toxics-3570961-supplementary.pdf]

Biogeochemical Mechanisms of HCO_3–Ca Water and NO_3^- Pollution in a Typical Piedmont Agricultural Area：Insights from Nitrification and Carbonate Weathering

Li Xu [1,2,†], Bo Xin [c], Wei Liu [2,†], Haoyang Liu [2], Guoli Yang [2,*], Guizhen Hao [2]

[1] *School of Energy and Environmental Engineering, Hebei University of Engineering, Handan 056038, China*

[2] *Hebei Key Laboratory of Water Quality Engineering and Comprehensive Utilization of Water Resources, Hebei University of Architecture, Zhangjiakou 075000, China*

[3] *Zhangcheng Ecological Environmental Protection and Restoration Technology Innovation Center, No.3 Geological Brigade of Hebei Geology and Mineral Exploration Bureau, Zhangjiakou 075000, China*

*Corresponding author email: YGL2004@163.com (Guoli Yang)

† These authors contributed equally to this work

Supplemental material

Including:

Table S1—Table S2;

Figure S1—Figure S5.

Text S1

Table S1.

Analytical methods for assessing chemical parameters and stable isotopes in river water samples in the study area.

Chemical compositions	Analytical methods	Testing institution
pH, DO, T, Eh, and TDS	HACH HQ40d multiparameter instrument (United States)	Hebei Key Laboratory of Water Quality Engineering and Comprehensive Utilization of Water Resources
K^+, Na^+, Ca^{2+}, and Mg^{2+}	Diane ICS 1500 ion chromatograph (United States)	Analysis and Testing Center, No.8 Institute of Geology and Mineral Resources Exploration of Shandong Province
Cl^-, SO_4^{2-}, and F^-	Vantone MIC ion chromatograph (Switzerland)	
HCO_3^-	Acid–base titration method	
NO_3^-, NO_2^-, and NH_4^+	Perkin-Elmer Lambda 35 spectrophotometry (United States)	
δ^2H-H_2O and $\delta^{18}O$-H_2O	Thermo Fisher Scientific GasBench II (United States) combined with Thermo Fisher Scientific MAT-253 (United States)	Key Laboratory of Groundwater Science and Engineering of the Ministry of Natural Resources
$\delta^{13}C$-DIC	Thermo Fisher Scientific Delta V Advantage (United States) combined with CTC Analytics GasBenchII (Switzerland)	Analysis and Testing Center, Third Institute of Oceanography, Ministry of Natural Resources

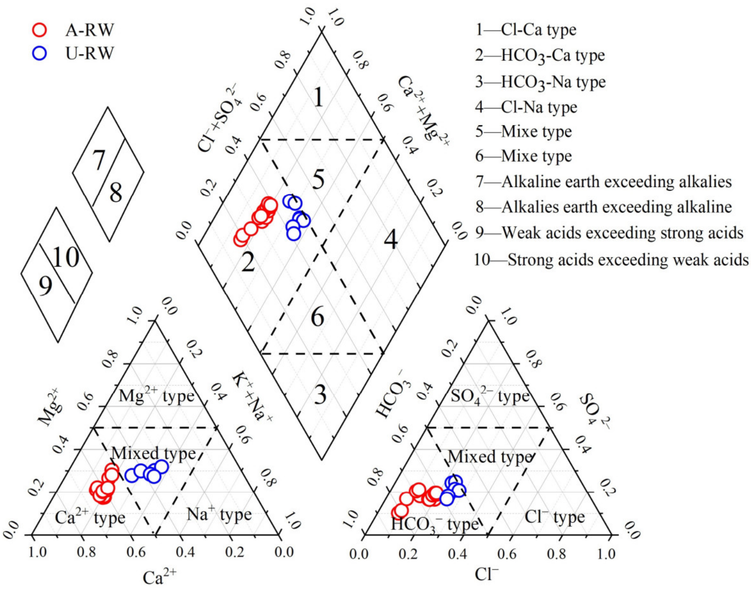

Figure S1. Piper diagram showing the hydrochemical facies and dominant cations and anions of river water in the up- (A-RW) and downstream (U-RW) regions of the Qingshui River.

A-RW (upper triangle); U-RW (lower triangle)

	pH	DO	ORP	TDS	K^+	Na^+	Ca^{2+}	Mg^{2+}	Cl^-	SO_4^{2-}	HCO_3^-	NO_3^-	NH_4^+
pH	1	0.15	-0.97	0.14	-0.072	0.12	-0.099	0.25	0.53	0.51	-0.49	0.59	-0.39
DO	0.95	1	-0.13	-0.027	0.24	-0.055	-0.33	-0.055	0.46	-0.082	-0.31	-0.21	-0.077
ORP	-0.99	-0.96	1	-0.099	0.088	-0.093	0.077	-0.2	-0.49	-0.4	0.47	-0.57	0.39
TDS	0.018	-0.036	0	1	-0.19	0.9	0.83	0.49	0.23	0.61	0.59	0.54	0
K^+	-0.36	-0.54	0.43	-0.25	1	0.022	-0.46	-0.8	0.54	-0.21	-0.58	0.12	0.46
Na^+	0.11	-0.14	0	0.071	0.79	1	0.64	0.34	0.29	0.65	0.44	0.51	0.23
Ca^{2+}	-0.18	-0.25	0.14	0.82	-0.29	-0.14	1	0.46	-0.16	0.27	0.82	0.45	0
Mg^{2+}	0.18	0.071	-0.14	0.93	-0.11	0.29	0.68	1	-0.27	0.6	0.55	0.038	-0.39
Cl^-	0.18	0	-0.11	0.71	0.36	0.68	0.39	0.86	1	0.18	-0.58	0.55	0
SO_4^{2-}	0.38	0.43	-0.39	0.82	-0.68	-0.29	0.61	0.79	0.43	1	0.17	0.37	-0.23
HCO_3^-	-0.52	-0.72	0.58	0.49	0.59	0.61	0.52	0.49	0.61	-0.072	1	-0.066	0
NO_3^-	-0.32	-0.15	0.22	-0.19	-0.11	-0.59	0.037	-0.41	-0.41	-0.15	-0.3	1	-0.15
NH_4^+	0.16	0.09	-0.14	0.56	0.18	0.31	0.38	0.47	0.65	0.31	0.32	0.26	1

Figure S2. Correlation analysis of physico-chemical data in the up- (A-RW; upper triangle) and

downstream (U-RW; lower triangle) regions of the Qingshui River.

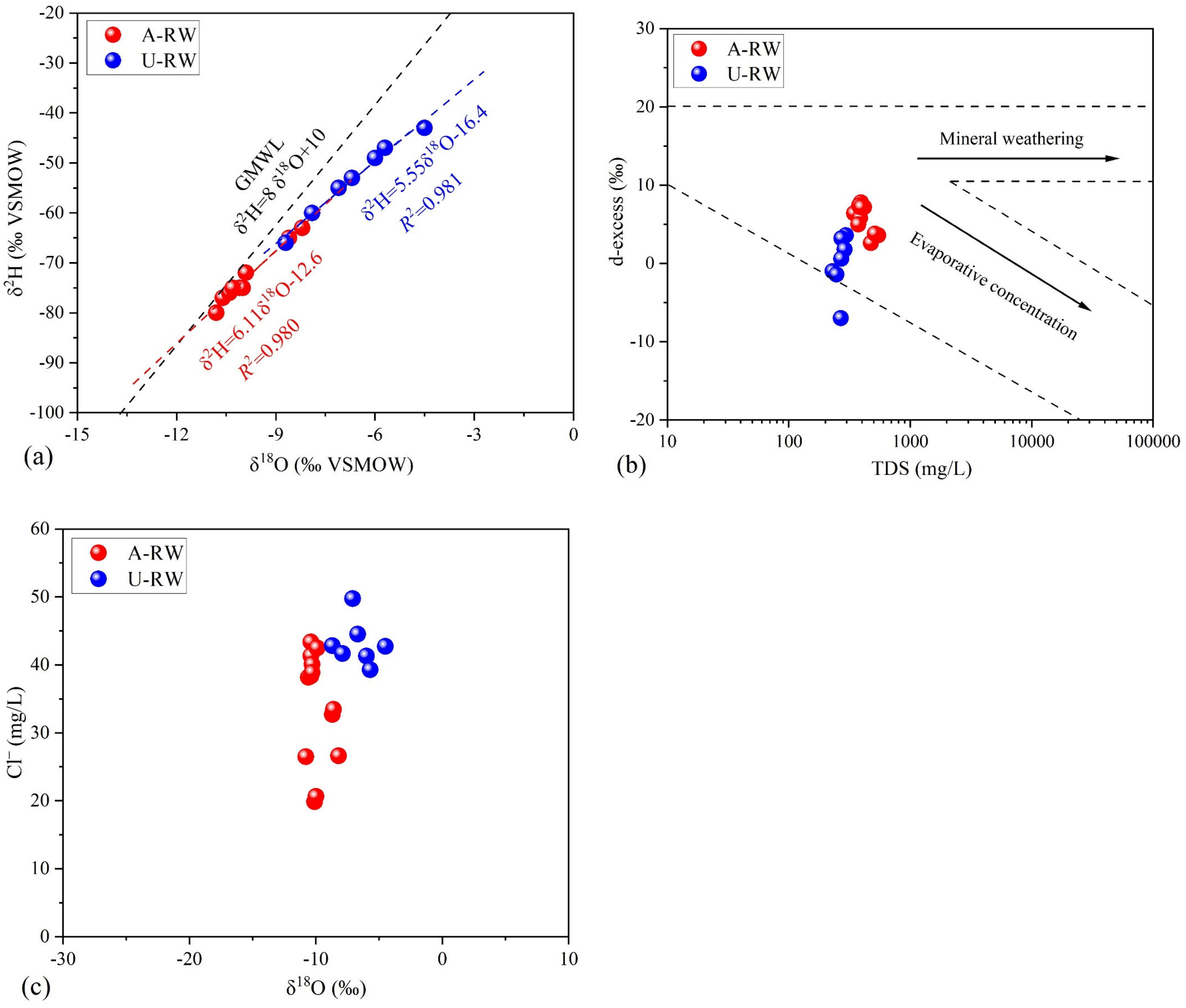

Figure S3. Diagrams of (a) $\delta^{18}O$-H_2O versus $\delta^{2}H$-H_2O, (b) TDS versus *d*-excess, and (c) $\delta^{18}O$-H_2O versus Cl^- in the up- (A-RW) and downstream (U-RW) of the Qingshui River. Note: the global meteoric water line (GMWL) was taken from Craig (1961).

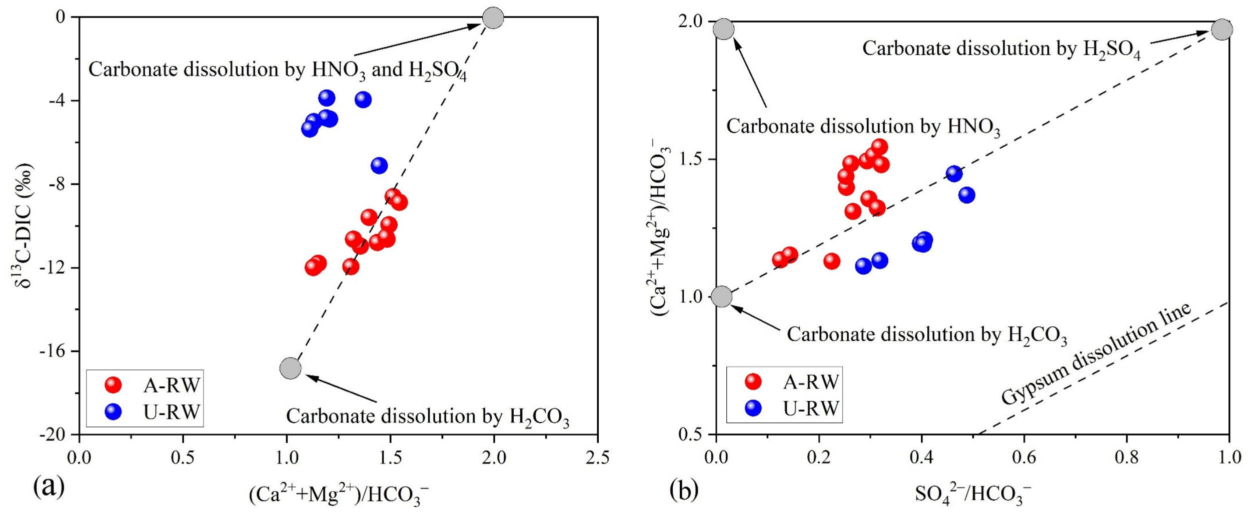

Figure S4. Involvement of strong acids in carbonate dissolution, as interpreted by scatter plot of (a) $(Ca^{2+}+Mg^{2+})/HCO_3^-$ versus $\delta^{13}C$-DIC (adapted from Xie et al., 2021) and (b) SO_4^{2-}/HCO_3^- versus $(Ca^{2+}+Mg^{2+})/HCO_3^-$ (adapted from Liu and Han, 2020) in the up- (A-RW) and downstream (U-RW) regions of the Qingshui River.

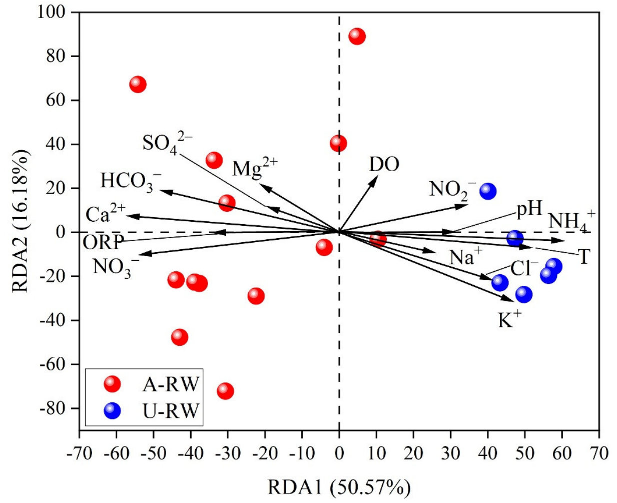

Figure S5. RDA analysis illustrating the effects of physico-chemical factors on microbial community structure.

Text S1 Analysis on the joint use of FM and SI to evaluate the driving mechanism of river hydrochemistry [63,64]

The calculation principle and sequence of the FM have been shown in detail in previous reports (Noh et al., 2009; Chen et al., 2021; Li et al., 2023). In short, the contribution ratio of mineral dissolution to hydrochemical compositions was calculated according to the mass budget equations of the dissolved solutes. The main minerals (i.e., carbonate, halite, gypsum and silicate) were considered in the FM. Considering the arid/semi-arid climate of the Qingshui River, precipitation (360-500 mm) presents a non-negligible source of dissolved solutes in river water. Thus, it was assumed that the content of dissolved solute X in river water (X_{Rw}) was derived from a combination of precipitation input (X_{Pre}) and halite (X_{Hal}), gypsum (X_{Gyp}), carbonate (X_{Car}) and silicate dissolutions (X_{Sil}), as described in Figure 2 and Eq. S4, as follows:

$$X_{Rw}=X_{Pre}+X_{Hal}+X_{Gyp}+X_{Sil}+X_{Car} \quad \text{(S4)}$$

The SI is a key indicator, illustrating the thermodynamic equilibrium state of mineral dissolution (Wang et al., 2023). The SI levels for the dissolution of halite (SI_{Halite}), gypsum (SI_{Gypsum}), dolomite ($SI_{Dolomite}$), and calcite ($SI_{Calcite}$) were determined using the hydrogeochemical simulation software, PHREEQC (v. 2.12.5). When the SI value was < 0, = 0, or >0, the selected minerals were considered to be in undersaturated, equilibrium and supersaturated states, respectively (Zhu et al., 2020; He et al., 2022).

A total of 1,306,064 optimized sequences, with a mean length of 375 bp, were obtained from high-throughput sequencing analysis. Clustering was performed based on operational taxonomic unit similarities of > 97%; the ribosomal database project classifier (RDP; v. 11.5), coupled with the reference

database (Silva, v. 138), was used to perform microbial phylogenetic classification at the domain–species level. Differences in microbial community structure between the A-RW and U-RW were evaluated using a sample–species relationship diagram (Circos-0.67-7), Venn petal diagram (R software, v. 3.3.1) and hierarchical clustering tree (Qiime 2020.2.0 and R software v. 3.3.1). The influences of physico-chemical indicators on microbial communities were assessed using redundancy analysis (RDA; vegan package in R software, v. 2.4.3). The Spearman coefficient was used to determine the statistical matrix between the physico-chemical indicators and microbial composition (heatmap package in R software v. 3.3.1). The microbial functions were assessed by comparing the measured molecular–biological data with the FAPROTAX database (v. 1.2.1).

References

Craig, H., 1961. Isotopic variations in meteoric waters. Science 133, 1702–1703.

Dansgaard, W., 1964. Stable isotopes in precipitation. Tellus 16, 436–468.

Liu, J., Han, G., 2020. Effects of chemical weathering and CO_2 outgassing on $\delta^{13}C_{DIC}$ signals in a karst watershed. J. Hydrol. 589, 125192.

Xie, Y., Huang, F., Yang, H., Yu, S., 2021. Role of anthropogenic sulfuric and nitric acids in carbonate weathering and associated carbon sink budget in a karst catchment (Guohua), southwestern China. J. Hydrol. 599, 126287.

Noh, H., Huh, Y., Qin, J., Ellis, A., 2009. Chemical weathering in the Three Rivers region of Eastern Tibet. Geochim. Cosmochim. Acta 73, 1857–1877.

Chen, J., Gao, Y., Qian, H., Ren, W., Qu, W., 2021. Hydrogeochemical evidence for fluoride behavior in groundwater and the associated risk to human health for a large irrigation plain in the Yellow River

Basin. Sci. Total Environ. 800, 149428.

Li, J., Zhou, Y., Zhou, J., Sun, Y., Zeng, Y., Ding, Q., 2023. Hydrogeochemical evidence for fluoride sources and enrichment in desert groundwater: a case study of Cherchen River Basin, northwestern China. J. Contam. Hydrol. 259, 104270.

Wang, S., Chen, J., Zhang, S., Zhang, X., Chen, D., Zhou, J., 2023. Hydrochemical evolution characteristics, controlling factors, and high nitrate hazards of shallow groundwater in a typical agricultural area of Nansi Lake Basin, North China. Environ. Res. 223, 115430.

Zhu, G., Wu, X., Ge, J., Liu, F., Zhao, W., Wu, C., 2020. Influence of mining activities on groundwater hydrochemistry and heavy metal migration using a self-organizing map (SOM). J. Clean. Prod. 257, 120664.

He, H., Wang, Y., Liu, Z., Bao, Q., Wei, Y., Chen, C., Sun, H., 2022. Lake metabolic processes and their effects on the carbonate weathering CO_2 sink: Insights from diel variations in the hydrochemistry of a typical karst lake in SW China. Water Res. 222, 118907.
